# Supplementary material for: TRPM8 thermosensation in poikilotherms mediates both skin colour and locomotor performance responses to cold temperature
Source: Commun Biol. 2023 Jan 31;6:127. doi: 10.1038/s42003-023-04489-8 (PMC9889708; doi:10.1038/s42003-023-04489-8)
Supplement: Supplementary file 2 — Supplementary Information [file 42003_2023_4489_MOESM2_ESM.pdf]

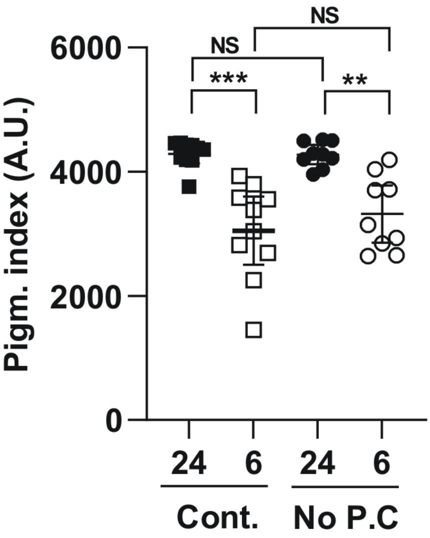

Supplementary Figure 1: Aggregation induced by cooling is independent of melatonin released by the pineal complex. Head pigmentation index of control (cont.) tadpoles or those pinealectomized 24 h before temperature treatment (No P.C.). Tadpoles were switched from 24 °C to 6 °C for 30 minutes. Each dot represented the measurement in one tadpole, and the bar is the mean with 95% confident interval; n=8 embryos; N=2 independent experiments; ** $p < 0.01$, *** $p < 0.001$; ANOVA followed by Bonferroni's test.

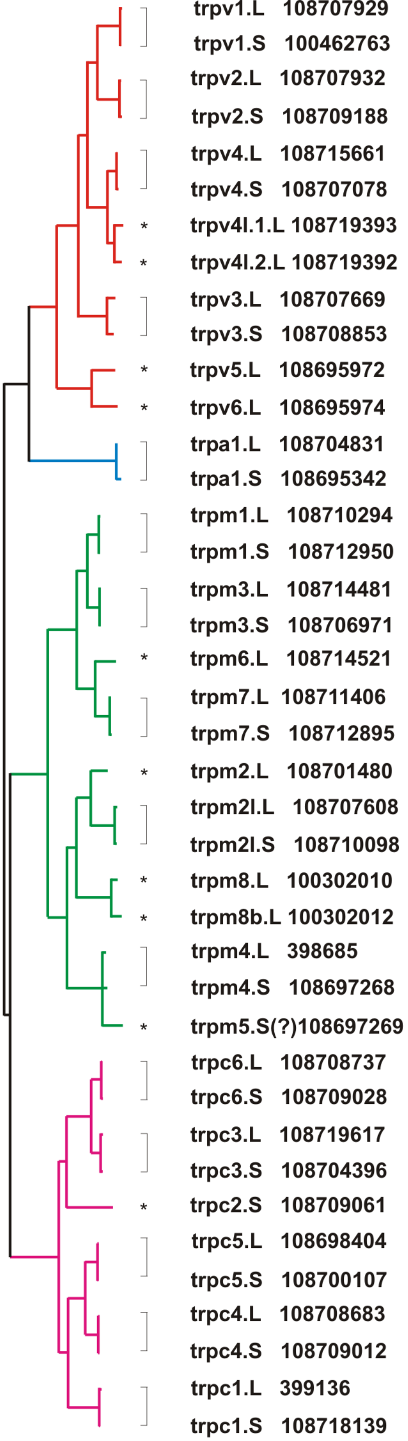

Supplementary Figure 2: Trp channels from group1 identified in X. laevis. A molecular phylogenetic analysis of group 1 family Xenopus laevis trp channel genes. The tree was performed by molecular phylogenetic analysis by the maximum-likelihood method using the public domain MEGA X version 10.04 software and is not drawn to scale. TRPV (red lines), TRPA (blue line), TRPM (green lines) and TRPC (purple lines) subfamilies are represented. Predicted amino acid sequences were aligned by MUSCLE. Phylogenetic analysis shows duplicated genes localized on the long (L) and short (S) homologous chromosomes of the allotetraploid species that remain conserved (brackets) [52], with other genes reduced to a single copy (asterisks). The gene numbers are indicated.

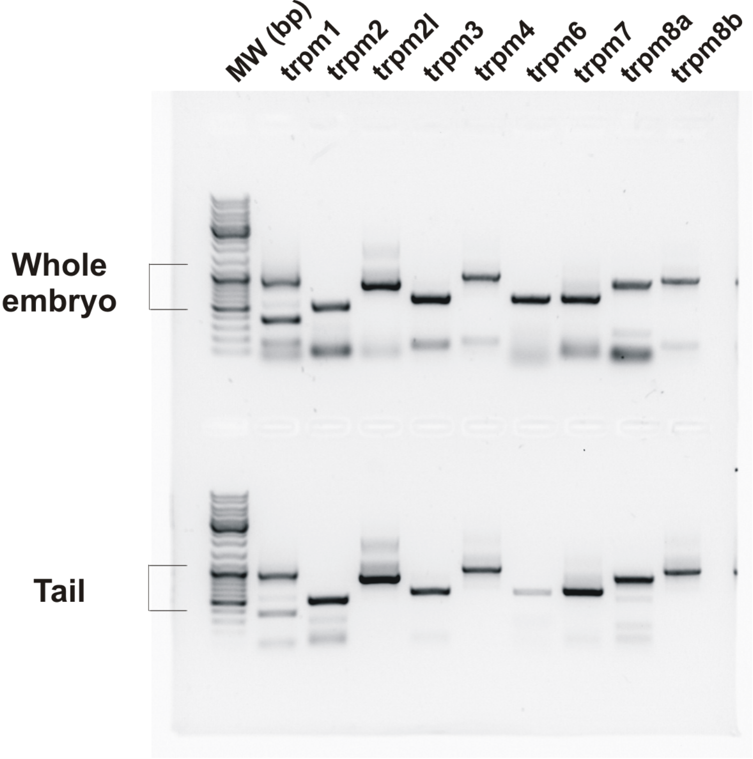

Supplementary Figure 3: Uncropped and unedited gel showing the RT-PCR analysis of mRNA expression for trpm1-trpm8b in the whole embryo (top) and tail (bottom) at stage 42/43. Indicated are the areas showed in Figure 3A.

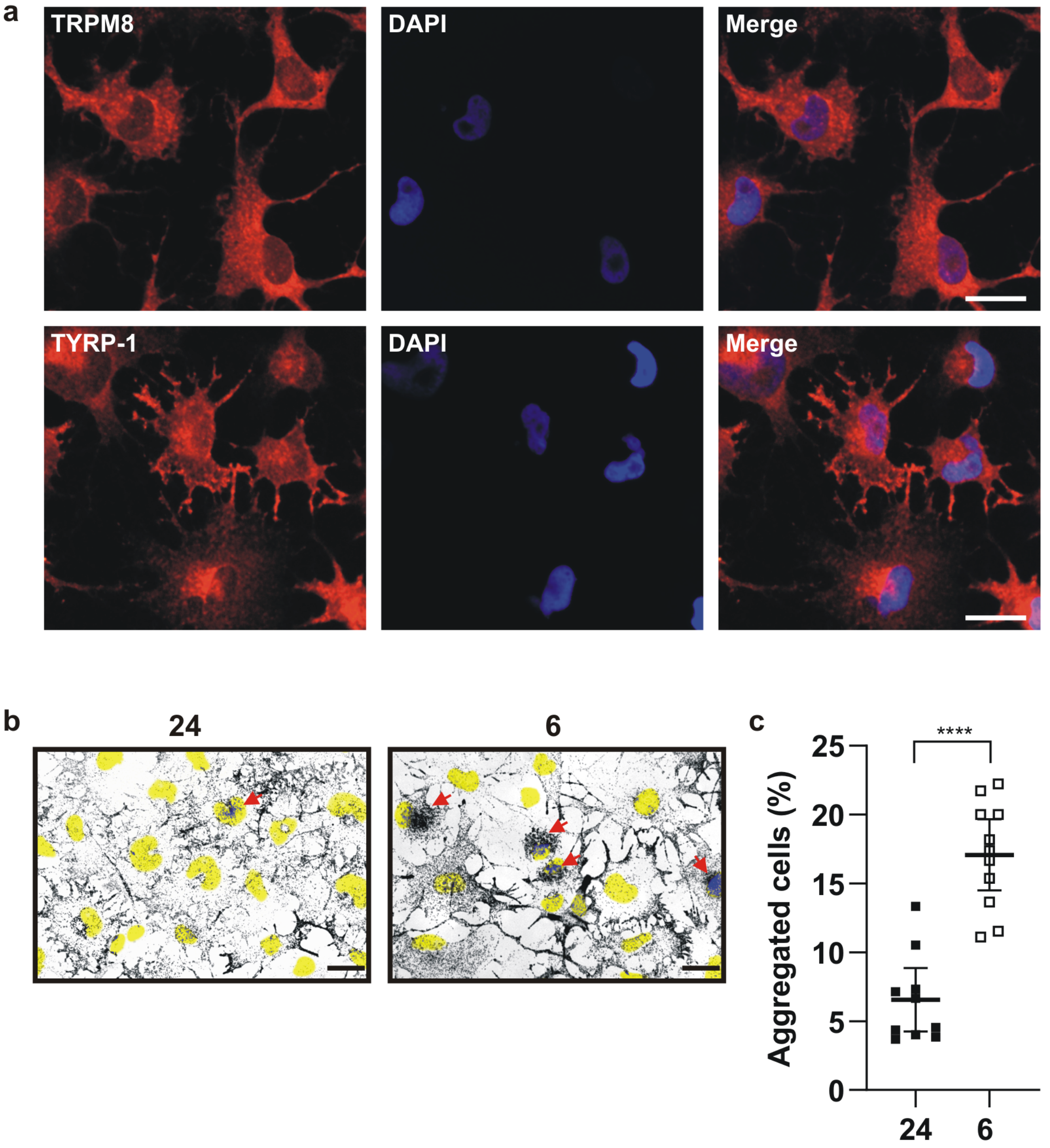

Supplementary Figure 4. Melanophore MEX cells express Trpm8 and aggregate melanosomes in cold conditions. a) Immunohistochemistry of a Xenopus melanophore cell line (MEX cells) with TRPM8 and TYRP-1 antibodies. Nuclei are stained with DAPI. Merge is shown. **b**) Phase-contrast microscopy merged with DAPI staining (yellow) of MEX cells incubated at 24 °C or 6 °C for 1 hour. Red arrows indicate melanosome aggregation. **c**) Percentage of cells with aggregated melanosomes per field (n=10 fields). Total cells counted: 17/260 and 46/237 at 24 and 6 °C, respectively. **** $p < 0.0001$; unpaired, Student's t-test. N=2 independent experiments. Scale bar = 10 μm

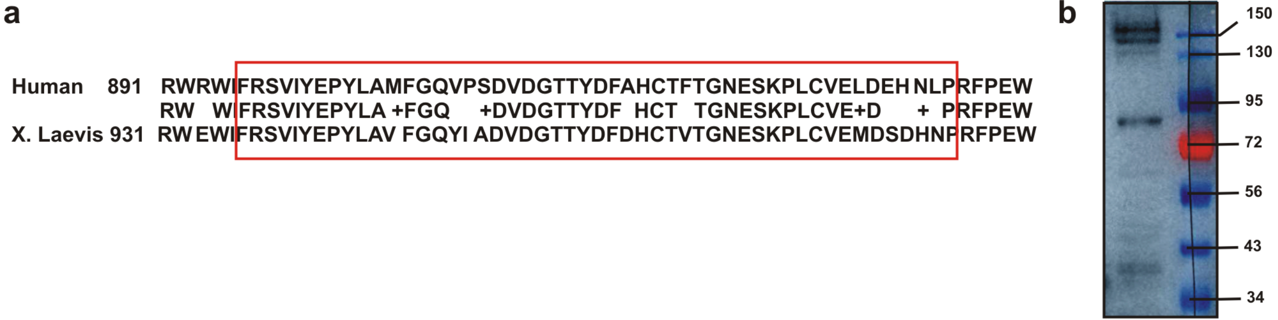

Supplementary Figure 5: Immunoreactivity against TRPM8. a) BLAST alignment illustrating similarity between the epitope used to generate an antibody against human TRPM8 (Human; (NP_076085.4) and the orthologous Trpm8a sequence in Xenopus (X. laevis; XP_018089453.1). The region recognized by the antibody is highlighted in red. **b)** Western blot of stage 42/43 Xenopus larvae with the anti-human TRPM8 antibody indicates doublet >130 kDa bands. Molecular markers (kDa) are indicated.

Supplementary Table 1. Trpm channels identified, and partial sequences cloned

Name	Model Entrez gene ID	Predicted mRNA	Vector	Gene Bank Acc # (Seq. cloned)	bp cloned	McFarlane plasmid number
Trpa1.S	108695342	XM_018223798.1	pCRII Topo	ON156681	1063	#974
Trpm8.L	100302010	NM_001161594.2	pCRII Topo	ON380668	884	#975
Trpm7.L	108711406	NM_001191021.3	pCRII Topo	ON380669	650	#977
Trpm8b.L	100302012	NM_001161595.2	pCRII Topo	ON380670	1177	#978
Trpm1l	108710294	XM_018251436.1	pCRII Topo	ON380671	958	#981
Trpm2l	108707608	XM_018245598.1	pCRII Topo	ON380672	829	#982
Trpm3.L	108714481	XM_018258772.1	pCRII Topo	ON380673	606	#983
Trpm6.L	108714521	XM_018258825.1	pCRII Topo	ON380674	610	#984
Trpm2.L	108701480	XM_018236213.1	pCRII Topo	ON380675	895	#985
Trpm4.L	398685	XM_018226434.1	pCRII Topo	ON380676	987	#986
