# Supplementary material for: TRPM8 thermosensation in poikilotherms mediates both skin colour and locomotor performance responses to cold temperature
Source: Commun Biol. 2023 Jan 31;6:127. doi: 10.1038/s42003-023-04489-8 (PMC9889708; doi:10.1038/s42003-023-04489-8)
Supplement: Supplementary file 3 — Reporting Summary [file 42003_2023_4489_MOESM3_ESM.pdf]

nature portfolio

Corresponding author(s): Double blind per review submission COMMSBIO-22-1650A

Last updated by author(s): Dec 7/2022

Reporting Summary

Nature Portfolio wishes to improve the reproducibility of the work that we publish. This form provides structure for consistency and transparency in reporting. For further information on Nature Portfolio policies, see our Editorial Policies and the Editorial Policy Checklist.

Statistics

For all statistical analyses, confirm that the following items are present in the figure legend, table legend, main text, or Methods section.

n/a	Confirmed	
☐	☒	The exact sample size (n) for each experimental group/condition, given as a discrete number and unit of measurement
☐	☒	A statement on whether measurements were taken from distinct samples or whether the same sample was measured repeatedly
☐	☒	The statistical test(s) used AND whether they are one- or two-sided<br>*Only common tests should be described solely by name; describe more complex techniques in the Methods section.*
☐	☒	A description of all covariates tested
☐	☒	A description of any assumptions or corrections, such as tests of normality and adjustment for multiple comparisons
☐	☒	A full description of the statistical parameters including central tendency (e.g. means) or other basic estimates (e.g. regression coefficient) AND variation (e.g. standard deviation) or associated estimates of uncertainty (e.g. confidence intervals)
☐	☒	For null hypothesis testing, the test statistic (e.g. F, t, r) with confidence intervals, effect sizes, degrees of freedom and P value noted<br>*Give P values as exact values whenever suitable.*
☒	☐	For Bayesian analysis, information on the choice of priors and Markov chain Monte Carlo settings
☒	☐	For hierarchical and complex designs, identification of the appropriate level for tests and full reporting of outcomes
☐	☒	Estimates of effect sizes (e.g. Cohen's d, Pearson's r), indicating how they were calculated

Our web collection on statistics for biologists contains articles on many of the points above.

Software and code

Policy information about availability of computer code

Data collection	Pictures from the dorsal head were converted to binary white/black images using NIH ImageJ public domain software. The density of positive pixels was used to determine the pigmentation index. The free online software, Kinovea version 0.8.15, was used to track the movement of tadpoles to determine the locomotor performance.
Data analysis	The statistical significance ($p < 0.05$) between treatments was determined with GraphPad Prism 9 by using multiple ANOVA followed by Bonferroni's post hoc test or t-test ($n \geq 8$; $N \geq 3$).

For manuscripts utilizing custom algorithms or software that are central to the research but not yet described in published literature, software must be made available to editors and reviewers. We strongly encourage code deposition in a community repository (e.g. GitHub). See the Nature Portfolio guidelines for submitting code & software for further information.

Data

Policy information about availability of data

All manuscripts must include a data availability statement. This statement should provide the following information, where applicable:

- Accession codes, unique identifiers, or web links for publicly available datasets
- A description of any restrictions on data availability
- For clinical datasets or third party data, please ensure that the statement adheres to our policy

Data have been deposited in the Dryad repository site with an assigned unique digital object identifier (DOI): doi:10.5061/dryad.6q573n61f. Cloned sequences were deposited in Gene Bank with access numbers provided in Supplementary table 1

Human research participants

Policy information about studies involving human research participants and Sex and Gender in Research.

Reporting on sex and gender	N/A
Population characteristics	N/A
Recruitment	N/A
Ethics oversight	N/A

Note that full information on the approval of the study protocol must also be provided in the manuscript.

Field-specific reporting

Please select the one below that is the best fit for your research. If you are not sure, read the appropriate sections before making your selection.

☐ Life sciences ☐ Behavioural & social sciences ☒ Ecological, evolutionary & environmental sciences

For a reference copy of the document with all sections, see nature.com/documents/nr-reporting-summary-flat.pdf

Ecological, evolutionary & environmental sciences study design

All studies must disclose on these points even when the disclosure is negative.

Study description	Using Xenopus laevis as a model system, we study two responses in ectotherms that contribute to temperature regulation: 1) A skin coloration response where dorsal pigmentation of the tadpole is adjusted to the environmental temperature to change the light refractory conditions, and 2) A behavioural response where the locomotor performance of the organism aligns with the ambient temperature. We explore both the molecular thermosensors, and the relationship between skin colour change and the locomotor preference in response to an ethologically-relevant cold temperature (24 and 6 Celsius degrees).
Research sample	Data were collected in a laboratory using Xenopus laevis tadpoles at two developmental time, stage 42/43 and stage 45/46. For all experiments, sample size in each group (control or treated) was between 8 and 12 tadpoles (n≥8); The experiments were performed three times (N=3).
Sampling strategy	Twelve tadpoles were set in a 60 mm dish at 24 degrees for 24/48 h before exposure to cooling temperatures. Individuals with developmental malformations (e.g. twisted tails, edemas etc) were removed before data collection (skin pigmentation or locomotor performance). Thus a minimum of 8 tadpoles were analyzed.
Data collection	Data were collected by Mr. Hannan Malik and Dr. Gabriel Bertolesi. For skin pigmentation studies, pictures of the dorsal head were taken with identical conditions of light, exposure time and diaphragm aperture, and converted to binary white/black images using NIH ImageJ public domain software. Pigmentation level (arbitrary units) was automatically converted by the software. For locomotor performance, a tray containing 9-12 tadpoles set individually in 35 mm dishes were recorded for one minute, every 5 minute during a total time of 30 minutes. The free online software, Kinovea version 0.8.15, was used to track the movement (mm) of each tadpole.
Timing and spatial scale	Skin pigmentation data were obtained between November 2018 and September 2021, unless correlation with locomotor performance was required. Locomotor performance data were obtained between July 2021 and March 2022.
Data exclusions	Tadpoles showing developmental malformation (e.g. twisted tails, edemas etc) were excluded for data collection.
Reproducibility	Skin pigmentation level changes considerably between hatches, therefore a representative experiment from at least three independent experiments (hatches) is shown. Also common is variation in the locomotor performance between hatches. Importantly cooling the temperature produced the same trend in all the experiments.

Randomization	Tadpoles were randomly grouped.
Blinding	Pigmentation level provided by the densitometry of the head picture and the locomotor performance measured as the movement into the dish were generated automatically by the Image J and Kniovea software respectively, therefore blinding the samples was not necessary.

Did the study involve field work? ☐ Yes ☒ No

Reporting for specific materials, systems and methods

We require information from authors about some types of materials, experimental systems and methods used in many studies. Here, indicate whether each material, system or method listed is relevant to your study. If you are not sure if a list item applies to your research, read the appropriate section before selecting a response.

Materials & experimental systems

n/a	Involved in the study
☐	☒ Antibodies
☐	☒ Eukaryotic cell lines
☒	☐ Palaeontology and archaeology
☐	☒ Animals and other organisms
☒	☐ Clinical data
☒	☐ Dual use research of concern

Methods

n/a	Involved in the study
☒	☐ ChIP-seq
☒	☐ Flow cytometry
☒	☐ MRI-based neuroimaging

Antibodies

Antibodies used	rabbit polyclonal anti-human TRPM8 (1:200 dilution; antibodies-online.com, ABIN351227) and rabbit polyclonal against human Tyrp-1 (1:200 dilution; Thermo Scientific, IL; PA5-81909)
Validation	ABIN351227: Reactivity in Xenopus laevis by immunohistochemistry and Western blot (showed in supplementary figures). Lack of staining in the absence of primary antibody.<br>PA5-81909: Provided by the supplier. Advanced validation; This antibody was verified by relative expression to ensure that the antibody binds to the antigen stated

Eukaryotic cell lines

Policy information about cell lines and Sex and Gender in Research

Cell line source(s)	The melanophore (MEX) cell line was originally generated from stage 35 Xenopus laevis embryos (ref #40) at Dr. Validimir Rodionov's laboratory, University of Connecticut.
Authentication	Immunoreactivity against Tyrp-1 confirmed the nature of the melanophore cell line.
Mycoplasma contamination	Lack of mycoplasma contamination was confirmed by DAPI staining.
Commonly misidentified lines (See ICLAC register)	The cell line obtained from Xenopus melanophores (MEX) is not registered in ICLAC

Animals and other research organisms

Policy information about studies involving animals; ARRIVE guidelines recommended for reporting animal research, and Sex and Gender in Research

Laboratory animals	Xenopus laevis embryos at stage 42/43 and 45/46 were used. Embryos were staged according to Nieuwkoop and Faber using Xenbase (http://www.xenbase.org) RRID:SCR_003280).
Wild animals	N/A
Reporting on sex	N/A
Field-collected samples	N/A
Ethics oversight	The Animal Care and Use Committee, University of Calgary, approved procedures involving frogs and embryos, which was signed by Dr Derrick Rancourt.

Note that full information on the approval of the study protocol must also be provided in the manuscript.
